# Frontiers in Invertebrate Physiology—An Update to the Grand Challenge

**DOI:** 10.3389/fphys.2020.00186

**Published:** 2020-02-28

**Authors:** Sylvia Anton, Christophe Gadenne, Frédéric Marion-Poll

**Affiliations:** ^1^UMR IGEPP INRA, Agrocampus Ouest, Université Rennes 1, Angers, France; ^2^Evolution, Génomes, Comportement, Ecologie, CNRS, IRD, Univ Paris-Sud, Université Paris-Saclay, Gif-sur-Yvette, France; ^3^AgroParisTech, Université Paris-Saclay, Paris, France

**Keywords:** genomic basis of physiology, plasticity of invertebrate physiology, adaptation to anthropogenic changes, nematode, *Drosophila*, ecophysiology, comparative physiology

“Studying invertebrate physiology is an exciting domain, because it provides on one hand insight into general principles of animal physiology, utilizing models with different degrees of complexity and on the other hand it allows studying evolutionary adaptations to a multitude of different lifestyles. Environmental constraints and basic construction principles have led to an amazing variation of physiological solutions to breathe, to ingest and digest food, to reproduce and to communicate and all this on the basis of a wide range of anatomical construction principles. Therefore, a comparative approach to invertebrate physiology is extremely rich and can only be encouraged. The goal of Frontiers in Invertebrate Physiology is to cover a wide range of approaches from the molecular to the cellular, organismic, and even the population level. Studies on model and non-model organisms and on all aspects of physiology will be published, to provide a forum for exchange of recent advances in the field.

Invertebrates represent 95% of all living animal species. They have colonized all habitats on earth, including Polar regions, deserts, and seas. Their external skeleton (at least for some of the most prominent invertebrate groups) and their segmented central nervous system make them unique models to study developmental physiology (e.g., molting processes) and the gradual architectural evolution of their central nervous system, and the resulting neural and sensory physiology. Moreover, many invertebrate species are organized in very sophisticated societies, thus offering exciting challenges to study the physiology of intra- and inter-specific interactions and their adaptation to environmental constraints (Woodard et al., 2011).”

## Genomic Search for Invertebrate Physiological Functions

Ten years of Frontiers in Invertebrate Physiology with an increasing number of submissions every year, illustrate the great progress in general in invertebrate physiology, and more specifically in integrating molecular advances with physiological function. After the extremely ambitious i5k initiative launched in 2011, intending to sequence the genomes of 5,000 insect species within 5 years (Robinson et al., 2011), 155 insect genomes have now been annotated, around 400 genomes have been assembled and more than 1,200 projects have been registered in the NCBI database (Li et al., 2019).

In addition to widely used correlative approaches, linking variations in gene expression with phenotypic variations in animal behavior and physiology, large progress has been made in the development of gene knockout methods for both model and non-model organisms, such as RNAi techniques and the more recent CRISPR Cas9 system (Tijsterman et al., 2002; Sun et al., 2017). Whereas mutants with well-defined deficiencies have been used for physiological studies in model organisms like *Drosophila melanogaster* and *Caenorhabditis elegans* for more than 40 years (Corsi et al., 2015; Kaufman, 2017), these latter approaches have recently helped to identify general physiological mechanisms down to the cellular and molecular level across species (He et al., 2019), but also uncovered variability in physiological mechanisms between different organisms. A large spectrum of Research Topics in Frontiers in Invertebrate Physiology illustrates this type of progress in multiple invertebrate taxa. Linking genomic and physiological results with invertebrate behavior requires now high throughput behavioral experiments with challenging analysis methods, involving for example machine-learning algorithms, creating the new discipline of ethomics (Geissmann et al., 2017).

## Invertebrate Physiology and Human Activities

Another field, which has advanced importantly in the last decade is the understanding of physiological mechanisms in invertebrates with importance in human activity, such as agriculture, aquaculture, and the transmission of diseases, with the ultimate goal to control pest species or improve rearing conditions for organisms used for human and animal nutrition (Frontiers in Invertebrate Physiology Research Topics on cephalopods, insect-pathogen-plant interactions, tick-and tick-borne pathogens, insect immune systems, digestive enzymes, adaptation to environmental stresses,…). Especially the control of pest species, such as herbivores and disease vectors, urgently requires the development of alternative methods, due to important changes in legislation concerning chemical control measures all over the world (for example the restriction of the use of neonicotinoid insecticides by the European Food Safety Authority in 2018). “Studies of host-pathogen interactions, immunity, and the physiology of resistance will not only be important for food production, but also for improving public health. Although invertebrate species have been used for medical purposes for more than 4,000 years and as models for research and teaching since the end of the eighteenth century, the development of invertebrate models for e.g., neurodegenerative diseases” (i.e., fruitflies) and drug addiction is recent and in plain expansion (Sarkar et al., 2016; von Staaden and Huber, 2019). Major physiological mechanisms arose very early in animal evolution and have been highly conserved in spite of the amazing diversification of external morphology. Therefore, high throughput approaches required by modern interdisciplinary research, feasible in model invertebrates such as *D. melanogaster* and *C. elegans*, will provide a solid basis to solve important questions in vertebrate, including human and clinical physiology. “These recent developments will allow to reduce the use of mammals for medical research (e.g., drug development), important issue from an ethical and economic point of view.” Environmental risks and risks for human and animal health, as well as the development of resistance of many pest species lead to important challenges in the coming decade to discover new physiological targets for pest control. Among emerging ideas for alternative pest management, reverse chemical ecology proposes new specific targets in form of olfactory proteins for pest control or conservation biology (Zhu et al., 2017; Choo et al., 2018). Another emerging target for pest control is to manipulate their digestive enzymes (Zibae, 2012).

## Invertebrate Physiology and Animal and Human Nutrition

An interesting twist of our interest in pest insects is to consider them as a potential source of proteins and nutritive substances. Since the FAO report about entomophagy and its potential to contribute to feed animals and humans (van Huis et al., 2013), considerable efforts have been made to develop the production of a few insect species like *Tenebrio molitor* and *Hermetia illucens* at an industrial scale in Europe and in the rest of the world. While the first species is a pest studied in the laboratory since a long time, data about other species like *Hermetia illucens* are scant. There is thus a great demand of data concerning the nutrition and reproduction physiology of such species, together with data about how their food affects their nutritional quality, as well as how to keep them healthy and resistant against their own pathogens.

## Invertebrate Adaptation to Anthropogenic Environmental Changes

Anthropogenic environmental changes, such as climate change and pollution also open a whole new field of research in invertebrate physiology in a context of ecological and environmental questions. Ecophysiological approaches with recent advances include system biology approaches (using modeling) (Damos et al., 2018) and adaptations to stress conditions (Tang et al., 2017). Climate change and pollution have also recently been shown to change intra- and interspecific communication channels (Boullis et al., 2016; Fuentes et al., 2016; Jürgens and Bischoff, 2017). However, the field of ecophysiology is only at its beginning and major research efforts in this field are needed in the future.

## Neural Plasticity of Invertebrates

“Another issue, which has been increasing enormously during the last years, is the investigation of plasticity in the nervous control of physiological functions (Yamada et al., 2010). Adaptations to external and internal modifications in sensory and motor systems controlling different physiological functions become more and more evident. Detection of intra- and inter-specific stimuli is modified by e.g., experience and reproductive state (Iyengar et al., 2010).” The last 10 years have shown important advances in the investigation of mechanisms of plasticity in invertebrates (Pyza, 2013; Vafopoulou, 2014). “However, we are far from understanding the cellular mechanisms and signaling pathways involved in these forms of physiological plasticity. It will be important in the future to investigate the role of hormones, neuromodulators, and their mechanisms of action ideally in parallel in different invertebrate species both during development and in adult organisms. New biochemical techniques allow us to detect and measure minute traces of neuropeptides in single cells, whose roles are still largely unknown (Yew et al., 2009).” Another important point is the rapidly developing field of epigenetics, showing an important role of DNA methylation in adaptation processes (Gavery and Roberts, 2013).

All parts of the manuscript in quotation marks are taken from Anton et al. (2011).

## Author Contributions

SA, CG, and FM-P contributed equally to the writing of the manuscript.

### Conflict of Interest

The authors declare that the research was conducted in the absence of any commercial or financial relationships that could be construed as a potential conflict of interest.
